# Harnessing the vaginal microbiome: a new frontier in the prevention of sexually transmitted infections

**DOI:** 10.1017/erm.2026.10062

**Published:** 2026-07-02

**Authors:** Roghayeh Mohammadzadeh, Erta Rajabi, Rosull Saadoon Abbood, Raad N. Hasan, Tahereh Navidifar, Narjess Bostanghadiri

**Affiliations:** 1 Department of Microbiology and Virology, School of Medicine, Mashhad University of Medical Sciences, Mashhad, Iran; 2 Faculty of Medicine, Tehran University of Medical Sciences, Tehran, Iran; 3 Medical Laboratory Techniques department, College of Health and medical technology, University of Al-maarif, Anbar, Iraq; 4 Biotechnology and Environmental Center, University of Fallujah, Iraq; 5 Department of Basic Sciences, Shoushtar Faculty of Medical Sciences, Shoushtar, Islamic Republic of Iran; 6 Research Center for Clinical Virology, Tehran University of Medical Sciences, Tehran, Islamic Republic of Iran

**Keywords:** Lactobacillus, sexually transmitted infections (STIs), vaginal microbiome, vaginal microbiota transplants (VMTs), Vaginal dysbiosis, Probiotics

## Abstract

**Background:**

The vaginal microbiome plays a vital role in maintaining reproductive and urogenital health in females. In healthy women, it is dominated by Lactobacillus species, including *L. crispatus*, *L. gasseri*, *L. iners*, and *L.* *jensenii*. These species provide strong protection against sexually transmitted infections (STIs) and other gynecological diseases. They produce lactic acid, which helps maintain a low vaginal pH, supporting epithelial integrity, modulating the immune system, and preventing colonization by bacterial pathogens. Dysbiosis, or an imbalance in microbial communities, is linked to higher susceptibility to infections like *Chlamydia trachomatis*, *Neisseria gonorrhoeae*, *Trichomonas* *vaginalis*, HIV, and HPV. The vaginal microbiota is dynamic and can change in response to hormonal shifts, sexual activity, antibiotic use, hygiene practices, and the duration of infection.

**Methods:**

This review provides a thorough overview of the composition, functions, and protective mechanisms of a healthy vaginal microbiome. **Results:** Recent research has shown that microbiome-based therapies, such as probiotics, prebiotics, synbiotics, and vaginal microbiota transplantation, may help restore vaginal microbial balance. Additionally, introducing engineered strains like *L. jensenii* that produce anti-HIV proteins offers promising new approaches to HIV treatment.

**Conclusions:**

This review also emphasizes therapeutic strategies aimed at stabilizing the vaginal microbiota to lower STI risk.

## Graphical abstract

**Figure:**
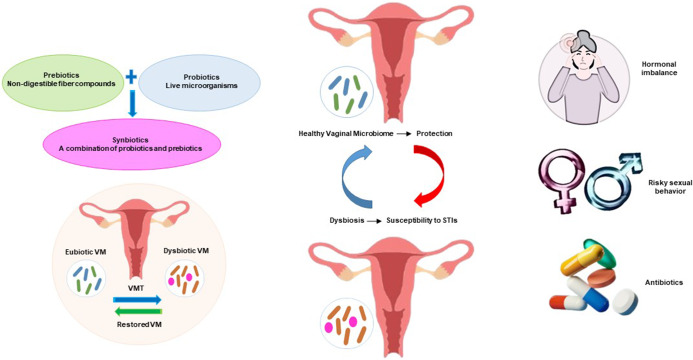

## Introduction

Sexually transmitted infections (STIs) represent a primary global health concern, with profound medical, social and economic consequences. These infections, caused by bacteria, viruses, and parasites, are primarily transmitted through sexual contact (Ref. 1). According to the World Health Organization, more than 1 million new STI cases occur every day worldwide. Among these, human immunodeficiency virus/acquired immunodeficiency syndrome (HIV/AIDS) remains a significant challenge, affecting over 38 million people globally (Ref. 2).

Bacterial STIs, such as chlamydia, gonorrhoea and syphilis, also continue to rise, and complications include infertility, ectopic pregnancy and adverse pregnancy outcomes. Additionally, affected individuals often suffer from stigma and emotional distress, which can delay diagnosis and treatment (Refs 1, 3).

One promising area of interest is the vaginal microbiome, which plays a critical role in maintaining reproductive and urogenital health. In healthy women, the vaginal microbiota is predominantly composed of Lactobacillus species, such as *Lactobacillus crispatus*, *L. gasseri*, *L. iners* and *L. jensenii* (Ref. 4). These bacteria contribute to vaginal homeostasis by producing lactic acid and hydrogen peroxide (H_2_O_2_), which lower vaginal pH, enhance epithelial barrier function, modulate immune responses and prevent colonization by pathogenic microbes.

Disruption of this microbial balance, a condition known as vaginal dysbiosis, increases susceptibility to a variety of STIs, including *Chlamydia trachomatis*, *Neisseria gonorrhoeae*, *Trichomonas vaginalis*, HIV and human papillomavirus (HPV). The vaginal microbiota is highly dynamic and influenced by numerous factors such as hormonal fluctuations, sexual activity, antibiotic use, personal hygiene practices and ageing (Refs 5, 6).

Conventional treatment of STIs typically involves antibiotics or antiviral drugs, depending on the pathogen type. However, the growing problem of antimicrobial resistance, particularly in organisms like *N. gonorrhoeae* and *T. pallidum*, has limited the effectiveness of current therapies (Ref. 7). This alarming trend calls for innovative solutions, including alternative therapeutic and preventative strategies (Ref. 8).

In recent years, microbiome-based therapies, including probiotics, prebiotics, synbiotics and vaginal microbiota transplantation (VMT), have emerged as novel approaches to restore vaginal microbial balance and reduce the risk of STIs (Ref. 9). Additionally, genetically engineered strains of Lactobacillus that produce antiviral proteins offer a new frontier in STI prevention, particularly against HIV (Ref. 10). Probiotics, primarily of the Lactobacillus genus, play a significant therapeutic role in combating sexually transmitted diseases (STDs). Their most significant health benefits include the production of lactic acid against pathogens through the modification of microbial surface proteins and the disruption of lipid membranes, resulting in cytosolic acidification and a redistribution of microbial metabolism. In addition, some *Lactobacillus* species produce and secrete H₂O₂ into the vaginal cavity and penetrate microbial membranes, and subsequently, an oxidative reaction occurs in the cytosol (Ref. 11).

VMT is considered a novel approach to the treatment of sexually transmitted diseases to re-establish homeostasis of the vaginal microbial community by transferring vaginal microbiota from a healthy donor to the vaginal cavity of another individual. Possible mechanisms of the therapeutic effects of VMT include increased microbial competition for access to nutrients and enhanced production of bacteriocins, virucidal substances and H₂O₂, as well as selective adhesion of beneficial microbes to the vaginal epithelium (Ref. 12).

Therefore, this review aims to provide a comprehensive overview of the composition and functions of the healthy vaginal microbiome, examine its role in preventing sexually transmitted infections and evaluate current and emerging microbiome-targeted therapies for maintaining and restoring vaginal health.

## Composition and function of the healthy vaginal microbiome

### Composition of a healthy vaginal microbiome

The vaginal microbiome (VM) is a diverse and dynamic microbial community, a composition that fluctuates throughout different stages of a woman’s life (Ref. 13). Lactobacillus, first discovered in 1892, was thought to be the sole microbial agent of the VM (Ref. 14). However, it was later established that VM is a complex structure, comprising various microbial community state types (CSTs) that alternate between different life periods, depending on different menstrual cycle stages (Refs 15, 16). Until now, five major CSTs have been identified, including CST-I, -II, -III and -V, dominated by *L. crispatus, L. gasseri, L. iners*, and *L. jensenii*, respectively, and non-*Lactobacillus* anaerobic microorganisms, including *Gardnerella*, *Prevotella* and *Atopobium*, comprise CST-IV (Table 1) (Refs 17, 18, 19).

**Table 1.** Overview of CSTs and their bacterial composition in the vaginal microbiome Table 1. long description.

CST-I		CST-II	CST-III		CST-IV				CST-V
CST-IA	CST-IB		CST-IIIA	CST-IIIB	A	B	C0	C1, C2, C3, C4	
Dominated by *L. crispatus*	Dominated by *L. crispatus*	Dominated by *Lactobacillus gasseri*	Dominated by *Lactobacillus iners*	Dominated by *L. iners*	Dominated by *Candidatus Lachnocurva vaginae*	Dominated by *Gardnerella vaginalis*	Dominated by *Prevotella*	High *Streptococcus* spp.	Dominated by *Lactobacillus jensenii*
					Moderate *G. vaginalis*	moderate *Atopobium vaginae*		High *Enterococcus* spp.	
Higher abundance of *L. crispatus*	Higher abundance of *Lactobacillus acidophilus*					low abundance of *Candidatus Lachnocurva vaginae*		High *Bifidobacterium* spp.	
								High *Staphylococcus* spp. isolates	
Lower abundance of *L. acidophilus* and *L. iners*	High abundance of *L. iners*			Co-dominated by *L. acidophilus* and non-*Lactobacillus* species	Moderate *A. vaginae*				
Lower diversity than CST-IB	Higher diversity than CST-IA								

CSTs: community state types.

In post-menopausal women, a shift from *Lactobacilli*-dominant CSTs to CST-IV is observed, and the VM composition of post-menopausal women is predominantly inhabited by *Streptococcus* spp. (CST-IV C1, C2, C3, and C4) and *Prevotella* spp. (CST-IV C0), while *Lactobacillus* species (CSTs-I to III) are the dominant species in premenopausal women, in contrast to *Atopobium* species (CSTs-IV A and B), which are the predominant bacteria in perimenopausal women (Refs 20, 21). Moreover, although the VM in most healthy women of reproductive age consists of CST-I to CST-III, approximately 25% of women of child-bearing age do not possess a *Lactobacillus*-dominant vaginal composition, as observed in African and Hispanic women, probably due to cultural, behavioural and genetic differences (Ref. 22).

Throughout the pregnancy, the abundance of *Faecalibacterium* declines, and an increase in *Proteobacteria* spp., *Streptococci* and some *Lactobacilli* species, including *L. vaginalis*, *L. crispatus*, *L. gasseri*, and *L. jensenii*, is more prominent, particularly within the third trimester, resulting in a less diverse microbial community, with *Lactobacillus* species being the dominant group (Refs 23, 24). Additionally, one longitudinal study of British women with uncomplicated pregnancies reported CST-I in 40%, CST-III in 27%, CST-V in 13%, CST-II in 9% and CST-IV in 8%. Nevertheless, not all women experience such alterations, as elevated levels of proteobacteria in the third trimester are not always documented in pregnant participants, possibly due to differences in gene expression profiles (Ref. 25).

### Protective mechanisms of the vaginal microbiota

**Figure 1.**
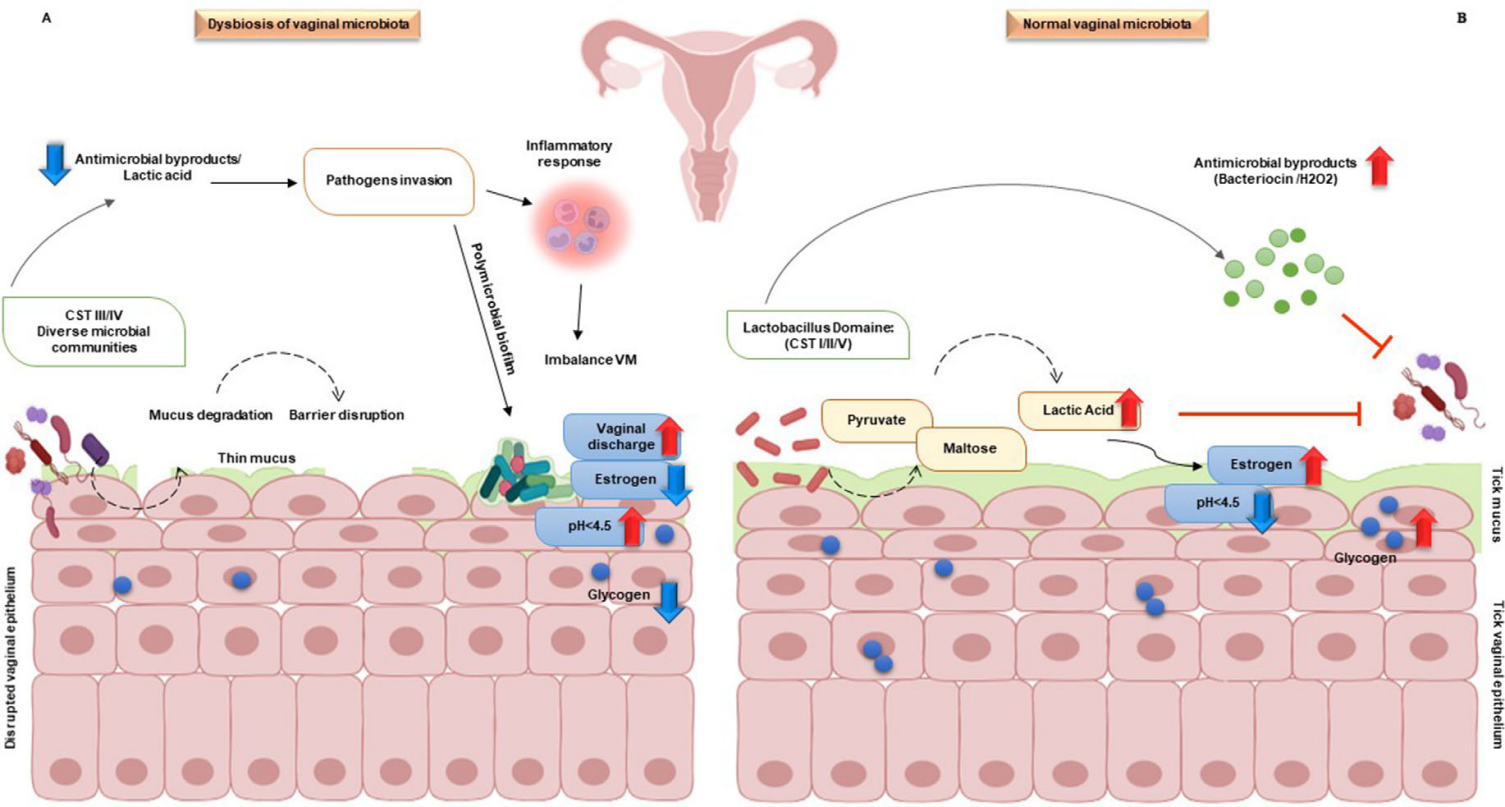
**A.** Dysbiosis of vaginal microbiota and mechanisms of pathogens’ virulence. **B.** Normal vaginal microbiota and the Lactobacillus mechanism of action. Figure 1. long description.

While CST-IV presence in a proportion of reproductive women is considered healthy, it has been associated with various vaginal-related infections (Ref. 26). To maintain VM composition, various defence mechanisms within the vaginal cavity have evolved (Figure 1).

### Lactic acid production

Lactic acid (LA), produced by *L. acidophilus*, contains two isoforms, including dextro and levo, and is capable of inhibiting several pathological microorganisms’ growth, including *chlamydial* growth inhibition at LA concentrations of 48–167 mM (Ref. 27). Gong et al. speculated that the inactivation of responsible particles for cell attachment and the reduction of disulfide in the pathogen’s membrane, leading to membrane disruption, were the primary mechanisms by which LA exerted its effects (Ref. 28). Most recently, another study documented suppression of tumour necrosis factor-α (TNF-α) and nuclear factor kappa-light-chain-enhancer of activated B cells (NF-κB) and enhancement of vaginal epithelial barrier integrity due to increased cell–cell adhesion protein expression in a dose- and pH-dependent manner at 100 mM concentrations of LA, proposing that there may be more to the role of LA in vaginal microbiome community balance and defence against microbiome dysbiosis (Ref. 29). Additionally, LA appears to irreversibly disrupt HIV infectivity at concentrations of 0.5% or higher, probably through unfolding of HIV glycoprotein (gp) 120 envelope protein (Ref. 30). Moreover, similar results to HIV have been demonstrated regarding herpes simplex virus (HSV), *C. trachomatis* and *N. gonorrhoeae* (Ref. 31). Furthermore, an increase in LA results in a lower vaginal pH, providing a suitable medium for *Lactobacillus* species development, thereby increasing H_2_O_2_ and bacteriocin production, which are known for their antimicrobial properties (Ref. 32).

### Hydrogen peroxide

H_2_O_2_ is an oxidizing, antimicrobial byproduct of vaginal microorganisms that is involved in various cell functions, including proliferation promotion and protein regulation (Ref. 33). This water-soluble ubiquitous molecule is synthesized within the vaginal cavity by anaerobic microorganisms, including *Lactobacilli*, which colonize as high as 10^6^–10^7^ organisms per mL (Ref. 34). Additionally, the NADH-flavin reductase has been hypothesized to have a role in H_2_O_2_ production in several *Lactobacillus* species deficient in H_2_O_2_-scavenging enzymes (Ref. 35). The oxidizing property of this molecule increases radical OH production, which in turn induces DNA damage in the invading pathogens (Ref. 36). Although H_2_O_2_ is efficient in the elimination of pathogenic microorganisms at physiological levels, higher concentrations induce *Lactobacillus* species destruction (Ref. 37). Moreover, evidence suggests that H_2_O_2_ functions as an immunomodulator within the vaginal environment rather than as an antimicrobial agent, as it promotes immune cell recruitment, suppresses inflammasome activation and regulates immune responses (Ref. 38). Moreover, despite speculations, physiological and supra-physiological levels of H_2_O_2_ have been reported to have no antimicrobial effects on pathogens responsible for BV, corroborated by Mijacˇ et al., who observed no protective effects in the presence of H_2_O_2_-producing *Lactobacilli*, as the abundance of such microorganisms was similar in women with vulvovaginal candidiasis (VVC) and women with normal flora (Refs 37, 39). Conversely, another study reported declining rates of *Candida* spp isolations following exposure to H_2_O_2_-producing *Lactobacilli* (Ref. 34). Altogether, the data support the hypothesis that H_2_O_2_ may exert antibacterial effects in the presence of other antimicrobial agents, whereas in isolation, its antibacterial properties are limited.

### Bacteriocin secretion

Bacteriocins are ribosomally synthesized, protease-sensitive peptides or small proteins produced by Gram-positive and Gram-negative bacteria, exhibiting both bactericidal and bacteriostatic activities, which, based on structural features, heat stability and post-translational modifications, are categorized into three major classes: (1) Class I comprises post-translationally modified bacteriocins, including lantibiotics (Ia), labyrinthopeptins (Ib) and sanctibiotics (Ic); (2) Class II consists of unmodified, heat-stable bacteriocins, divided into pediocin-like (IIa), two-peptide (IIb), circular (IIc) and linear (IId) types; and (3) Class III bacteriocins are heat sensitive, such as colicin from *Escherichia coli*, helveticin M from *L. crispatus*, helveticin J from *L. helveticus* and enterolysin A from *Enterococcus faecalis* (Ref. 40). Following their synthesis, bacteriocins attach to specific receptors on pathogenic microorganisms, permeabilize the bacterial cell wall and induce pore formation, eventually leading to cell leakage and intracellular adenosine triphosphate (ATP) depletion (Ref. 41). Additional effects include inhibiting intracellular enzymes by microcins and colicins, and cell wall degradation, particularly by lantibiotics and class IIa bacteriocins (Ref. 42). Moreover, class I imposes its antibacterial effect by inhibiting cell membrane synthesis, while class II exerts this effect through pore formation (Ref. 43).

Bacteriocins have also been reported to influence pathogenic bacteria within the VM community through mechanisms with inhibitory spectra ranging from broad to narrow and by preventing invasion by other species. One recent study reported in vitro identification of a bacteriocin produced by *L. acidophilus* KS400, which imposed its antimicrobial effects against *Gardnerella vaginalis*, *Streptococcus agalactiae* and *Pseudomonas aeruginosa*, with a loss in the antibacterial properties 32 hours post-fermentation, probably due to proteolytic degradation (Ref. 44). Moreover, bacteriocins produced by *L. salivarius*, *L. fermentum* and *L. paragasseri* are effective against *E. faecalis, Enterococcus faecium*, *N. gonorrhoeae*, and two *Candida* species (Refs 45, 46, 47).

### Competitive exclusion and biofilm formation

Although it has been speculated that the composition of biofilm microorganisms is formed through the cooperation of different species, it is now evident that such a structure is accomplished through competition within biofilms, which are a matrix assembly of living microorganisms (Ref. 48). In biofilms, microorganisms cooperate in synchrony against external threats via the Allee effect, which predicts a positive correlation between group density and composition, underscoring the benefits of grouping (Ref. 49). Although beneficial, such a composition increases the concentrations of antagonist metabolites, thereby increasing competition between microorganisms within the same matrix through suffocation, secretion of toxins into adjacent cells and antibiotic secretions against other species or even their kin, also known as kin competitors, which is observed in *P. aeruginosa* that exerts its competitive behaviour through two distinct strategies, bacteriophage utilization and changes in cyclic dimeric guanosine monophosphate (c-di-GMP), aiding surface adhesion (Refs 50, 51). Similar interactions within the vaginal microbiome community can be observed in both pathogenic and protective biofilms (formed by *Lactobacillus* species), including *G. vaginalis*, which is categorized into four subgroups (A, B, C and D), often found together within a biofilm and resulting in increased kin competition through contact-dependent ways, which involves direct inoculation of bacterial toxins into their competitors (Ref. 52). Although contact-independent interactions have been speculated to exist among *G. vaginalis* subgroups, recent studies do not support this notion and instead attribute *G. vaginalis* kin competition to contact-dependent mechanisms (Ref. 53). Moreover, *G. vaginalis* species appear to compete in a non-interfering, exploitative manner, known as scramble competition, in which the dominant subgroup (subgroup D) has the greatest ability to exploit a shared environment (Ref. 53). Additionally, *Lactobacilli* species, the critical strains in the vaginal microbiome, exert their competitive traits through competition for nutrients, production of bacteriocins, exopolysaccharides and H_2_O_2_ and co-aggregation, ultimately leading to biofilm composition domination and hindering pathogenic microorganism growth, similarly observed within one study, where *L. crispatus* and *L. plantarum* strains were reported to produce highly potent anti-Candida cell-free supernatants (CFSs), further emphasizing the competitive nature of Lactobacilli species due to the competition for nutrients and inhibition of *C. albicans* adhesion and biofilm formation (Ref. 54).

### Immune modulation and barrier reinforcement

*Lactobacilli* strains may serve as probiotics, which are living microorganisms that ‘confer a health benefit to the host’, and have been reported to significantly mitigate inflammatory responses by reducing TNF-α and IL-1β and increasing IL-10. This anti-inflammatory cytokine effectively regulates host immune responses, thereby reducing unnecessary inflammation (Ref. 55). *L. crispatus*, a key component of CST-I, has recently been demonstrated to decrease *Helicobacter pylori* adhesion to gastric epithelial cells and downregulate gene expression of several pro-inflammatory cytokines, including IL-1β, IL-6, IL-8 and TNF-α, in addition to upregulation of IL-10, and TGF-ß and avoidance of innate immune responses stimulation via inhibition of toll-like receptor 2 (TLR2) ligand activation by masking this ligand with S-layer proteins, which are known modulators of immune system and inflammatory responses, resulting in decreased TLR2-dependent pro-inflammatory pathway activation, and thereby modulating immune responses and protecting vaginal barrier against pathogen colonization (Refs 56, 57). Additionally, *L. crispatus* reduces *C. albicans* virulence and modulates cytokine production and cell adherence in vitro (Ref. 58). *L. gasseri*, similar to *L. crispatus*, suppresses disintegrin and metalloprotease 17 (also known as TNF-α-converting enzyme) expression, regulates the NF-κB pathway, a known factor of inflammation response induction, and inhibits pro-inflammatory cytokine production, such as IL-6, against *H. pylori* (Refs 59, 60). Consistently, this strain exerts immunomodulatory effects within the vaginal environment through indole-3-lactic acid production, which activates an aryl hydrocarbon receptor-mediated immune response (Ref. 61). Moreover, *L. iners* is primarily known for its immunomodulatory and barrier-enhancing effects due to its role in increased TNF mRNA expression and activation of TLR signalling pathways under stress, and similarly, *L. jensenii* inhibits NF-κB and MAPK signalling pathways and reduces IL-6, IL-1α and IL-8 production, in addition to increasing IL-1β, IL-12 and IL-10 production (Refs 60, 62, 63). Additionally, increased mucus viscosity induced by Lactobacillus confers anti-DNA damage effects and viral trapping, thereby enhancing the vaginal barrier (Refs 6, 64, 65).

### Vaginal microbiome dysbiosis

Vaginal dysbiosis (VD) is defined as any imbalance within the vaginal microbiome that results in disruption of normal *Lactobacillus* dominance, the hallmark of vaginal health, and overgrowth of pathogenic microorganisms, such as anaerobic and facultative bacteria, including *C. albicans*, *T. vaginalis*, *N. gonorrhoeae*, *Mycoplasma genitalium*, *C. trachomatis* and viral pathogens, with a global prevalence of 18%, most commonly presenting as BV, which has been associated with various other infections, including HPV, HIV, *C. trachomatis*, and *N. gonorrhoeae* infections (Refs 66, 67). VD increases the risk of gynaecological non-infectious disorders as well, including cervical cancer, preterm birth and infertility, miscarriages, intrauterine adhesions, polycystic ovary syndrome and uterine fibroids (Refs 68, 69). Known factors contributing to VD are sexual activity, antibiotic use, hormonal fluctuations, immunosuppression, lack of personal hygiene, ageing and lifestyle factors (Refs 24, 26).

In cases of vaginal microbiota disturbances, reduced *Lactobacilli* species result in alkaline pH (>4.5) and create an optimal environment for overgrowth of pathological microorganisms, resulting in biofilm formation and activation of the mammalian target of rapamycin (mTOR) pathway (Refs 70, 71). The mTOR pathway disrupts the function of epithelial cells and increases mucosal inflammation by promoting the production of pro-inflammatory cytokines, recruiting immune cells to the site of infection, and ultimately increasing vulnerability and the risk of persistent infection (Refs 72, 73). Additionally, *C. albicans* and *T. vaginalis* can enter the superficial layer of the vaginal lining and trigger an inflammatory response, which is characterized by polymorphonuclear cells and macrophages, inducing candidiasis presentations and dysbiosis (Ref. 74).

## Dysbiosis and increased risk of STI pathogens

### Human immunodeficiency virus (HIV)

Researchers have speculated that the inflammation and decreased epithelial integrity induced by BV can facilitate HIV entry into the tissue and increase NOD-, LRR-, and pyrin domain-containing protein 3 (NLRP3) inflammasome activation and pro-inflammatory cytokines, thereby increasing intracellular HIV expression (Ref. 75). *G. vaginalis* isolates, induced by BV, adhere to the vaginal epithelial cells and form biofilm and vaginolysin. This bacterial cholesterol-dependent toxin forms pores in the epithelium and triggers signalling cascades, thereby increasing lytic cell death (Ref. 70). Several BV-associated metabolites reduce anti-inflammatory cytokine production, including IL-8 and IL-1α, and increase the activation of the NLRP3 inflammasome, which in turn activates caspase-1 and triggers pyroptosis and production of pro-inflammatory cytokines, including IL-1β and IL-18, further amplified by the production of lipopolysaccharides via anaerobes (Ref. 76). One study specifically aimed to evaluate whether there is a difference between BV-associated bacterial species regarding genital HIV shedding and reported that women with lower *Lactobacilli* spp. and higher *M. hominis* isolates were at a 100-fold higher risk of genital HIV shedding (Ref. 77). Moreover, BV-positive women aged 35 years or older appear to be at higher risk of HIV seroconversion (Ref. 78).

Reduced *Lactobacillus* species abundance increases recruitment of immune cells, including CD4+ T-cells, which are HIV’s primary targets (Ref. 79). Interestingly, studies suggest that CD4+ T-cells and T-helper cells (Th) with higher CCR5 and α4β7 expression are more affected by HIV (Refs 80, 81). Notably, within the vaginal microbiome, Th17 CD4+ T-cells with the capability to produce IL-17A highly express both CCR5 and α4β7, which turns them into optimal targets for HIV (Ref. 80). Moreover, tissue-resident memory CD4+ T cells (T_RM), which usually reside in the lower female genital tract, are the main reservoirs for this viral pathogen (Ref. 82). Both of these CD4+ T-cell types are recruited in VD and potentially increase HIV acquisition in individuals with BV (Ref. 83).

#### C. trachomatis

*C. trachomatis* is a Gram-negative, obligate intracellular pathogen that takes the two forms of elementary body (EB), the infectious extracellular form, and reticulate body (RB), the non-infectious intracellular form, which, if left untreated, can ascend to the upper genital tract, leading to pelvic inflammatory disease, ectopic pregnancy, infertility and pelvic pain (Ref. 84). Upon transmission, EBs adhere to the host epithelial cells and form an intracellular inclusion that expands under the influence of effector proteins and facilitates EBs’ differentiation into active RBs that replicate intracellularly and redifferentiate into infectious EBs, initiating another cycle of infection (Ref. 85).

One of the main components of the vaginal physical barrier is the mucinoid fluid that traps microorganisms, characteristics of which can be altered upon infection with *C. trachomatis* (Ref. 86). Additionally, several studies have reported reductions in *C. trachomatis* abundance upon exposure to *Lactobacilli* species, mainly attributed to lactic acid production by *Lactobacilli* species, particularly D (−) lactic acid, resulting in an acidic environment that is unsupportive for *C. trachomatis* development, particularly observed in *the presence of L. crispatus* (Ref. 87). Such an effect can also be observed post infection, as inoculation of *Lactobacilli* species reduced EB shedding duration and chlamydia-induced cytokine production in one study (Ref. 31). Additionally, *Lactobacilli* species deplete the vaginal microenvironment of essential nutrients for *C. trachomatis* growth, hindering its replication (Ref. 31). On the contrary, an increase in *G. gingivalis* species, as observed in BV, results in increased sialidases and vaginolysin, which degrade the mucinoid fluid of the vagina, in addition to the increase in cleavage of E-cadherin and Desmoglein-1, activation of the NF-κB signalling pathway and the initiation of the cascade of pro-inflammatory cytokine production, all of which increase epithelial permeability and facilitate *C. trachomatis* growth (Refs 88, 89).

#### N. gonorrhoeae

*N. gonorrhoeae* is an aerobic microorganism that only inhabits humans, with annual global cases as high as 78 million, and colonization within various organs, including the ocular, genital, rectal and nasopharyngeal mucosa (Ref. 90). Upon transmission through sexual contact, *N. gonorrhoeae* adheres to epithelial cells and initiates colonization and replication in the presence of adequate nutrients, in addition to the invasion of non-ciliated cervical cells, resulting in the activation of the innate immune system activation and *N. gonorrhoea*’s opsonization (Refs 91, 92). Despite high sensitivity to the animals’ complement system, *N. gonorrhoeae* can escape the human complement system, possibly attributed to its ability to attach to C3b and create iC3b, the inactivated C3b, thus shielding itself from complement system recognition and opsonization (Ref. 93). Several studies have reported increased STIs, including *N. gonorrhoeae*, in the presence of cervicovaginal dysbiosis, including one study that reported increased symptomatic BV incidence in women colonized with BV-associated bacteria (Ref. 94). Additionally, in the absence of co-infection, *N. gonorrhoeae* occurred more in those with CST-III (*L. iners*-dominated CST) rather than *L. cripatus*, further confirming the speculation that D (−) lactic acid is the primary defence mechanism against *N. gonorrhoeae*, probably due to the higher pro-inflammatory cytokines and increased inflammation (Ref. 95). Vaginal pH ≤ 4.5 is among the primary components of the vaginal defence mechanism, which results in a suboptimal environment for the growth of BV-associated bacteria and *N. gonorrhoeae*, for which a pH of 5 or higher is considered suitable (Ref. 96). Additionally, although *N. gonorrhoeae* is believed to have an acid tolerance system, the short period between adhesion to epithelial cells and cellular invasion prevents the activation of such a system (Ref. 97). Moreover, the risk of gonococcal infection is reported to increase during active menstruation, probably due to the increased vaginal pH by 2 units during this period and the availability of iron, the nutrient source for *N. gonorrhoeae* (Ref. 98).

### Human papillomavirus (HPV)

HPVs are intracellular pathogens that infect the primitive keratinocytes of basal cells and enter the phase of viral DNA replication, leading to an increased number of viral copies per cell (50 to 100 copies per cell), and then enter deeper levels of the epithelial cell, where high-level viral gene expression occurs (Refs 99, 100). Moreover, HPV-positive individuals possess either CST-III VM, dominated by *L. iners* (CST-III), or lower *Lactobacilli* isolates compared to HPV-negative women, and BV has been associated with persistent HPV infection, as persistent inflammation chronically increases pro-inflammatory cytokines and reduces immune system’s ability to recognize and disable HPV, further increasing viral replication and invasion and thereby persistent HPV infection in the setting of vaginal dysbiosis (Refs 101, 102, 103, 104).

The vaginal epithelium is a stratified squamous epithelium, comprising various cells, including keratinocytes, one of the primary target cells in HPV infections that aid modulation of cell stress and identification of pathogens (Ref. 105). Particularly, such regulation is exerted through the expression of TLRs following TLR3 activation, including TLR7, and the initiation of the production of type I interferons (IFN), such as IFN-α and IFN-β, that possess antimicrobial properties and amplify Th1-type cytotoxic responses (Ref. 106). High-risk HPV species downregulate the production of IFNs via interfering with IFN gene expression and hinder activation of IFN-α-mediated signalling (Ref. 107). Additionally, stimulation of vaginal epithelial keratinocytes due to injury or infection increases NLRP3 inflammasome, which triggers pro-inflammatory cytokine production, including IL-1*β*, IL-1α, and IL-1, a pivotal cytokine in innate immune system activation that is responsible for activation of T helpers and dendritic cells and promotion of B cell maturation (Ref. 108). Moreover, recent research suggests that HPV downregulates pro-inflammatory cytokine gene expression, particularly IL-1*β* and IL-6, ultimately leading to the absence of signals necessary for recruitment of immune cells to the site of infection and resulting in long periods of unrecognized viral replication (Ref. 109). Expectedly, the high rates of uninterrupted viral replication can increase the risk of genomic instability, DNA damage, and neoplastic transformations (Ref. 110). Additionally, during the initial phases of HPV infection, low concentrations of antigens are secreted, which prevent the immune system from detecting the infection (Ref. 111). These mechanisms all lead to reduced vaginal defence against HPV and increase the rates of HPV escape from immune system recognition (Ref. 112).

### T. vaginalis

One of the most prevalent forms of STIs is trichomoniasis, which accounts for 250 million cases globally and is caused by an anaerobic extracellular protozoan parasite known as *T. vaginalis* (Ref. 113). Recent studies depict that *T. vaginalis* may have an association with vaginal microbiome, implicating that 72% of women with *T. vaginalis* possess CST-IV, particularly due to higher vaginal pH in the presence of CST-IV, and that 30% of *T. vaginalis*-infected women are co-infected with BV, with no apparent explanation on whether BV contributes to *T. vaginalis* pathogenesis or vice versa (Ref. 114). Additionally, the concurrent existence of CST-IV and *T. vaginalis* appears to disrupt epithelial tight junctions and increase epithelial permeability (Ref. 115).

Upon adherence to vaginal epithelial cells via parasite surface lipoglycan and other membrane proteins, *T. vaginalis* induces the production of pro-inflammatory cytokines, including IL-1, IL-6, IL-8, and IL-17 (Ref. 116). IL-1, in particular, can activate the NLRP3 inflammasome, resulting in an activated NF-κB pathway and increased pyroptosis, thereby creating a vicious cycle of vaginal dysbiosis and increased inflammation that is triggered via reductions in *Lactobacilli* species abundance, resulting in increased vaginal pH, elevated pro-inflammatory cytokine production, degradation of vaginal mucosa, increased cell death and increased growth of anaerobic bacteria, which in turn further increase the inflammation and promote growth of *T. vaginalis* (Ref. 117). Moreover, existing literature suggests that *C. albicans* and *M. hominis* can amplify the cytopathic properties of *T. vaginalis* in cases of co-infection (Refs 118, 119). A recent systematic review and meta-analysis indicated an odds ratio (OR) of 1.87 and a hazard ratio (HR) of 2.08 regarding the association between BV and *T. vaginalis* co-infection. This is further corroborated by another study that found a two-fold increased risk of *T. vaginalis* infection acquisition in women with BV, suggesting a potential link to the diminished abundance of Lactobacilli caused by BV (Ref. 120).

## Factors influencing the vaginal microbiome

### Hormonal contraception and menopause

VM colonization is traditionally thought to occur at birth when newborns inherit either maternal or environmental microbiota through vaginal delivery or C-section. However, recent studies suggest that colonization may initiate during the foetal stage, as few studies have reported isolating microorganisms from amniotic fluid and other foetal tissues (Ref. 121). From birth until around 10 years of age, when the ovaries begin to produce oestrogen, a low abundance of *Lactobacilli* species has been reported (Ref. 122). Following increased oestrogen production, an increase in glycogen and thickness of epithelium is observed. Furthermore, increased progesterone production during the luteal phase can promote epithelial turnover and shedding, leading to the release of glycogen from desquamated epithelial cells into the vaginal lumen. This free glycogen serves as a primary substrate for vaginal microbes, which metabolize it into glucose and subsequently ferment it into lactic acid, resulting in a reduced vaginal pH, suggesting that hormonal contraceptives may also affect Lactobacillus colonization (Ref. 123). However, despite the protective role of lactic acid, menstruation can transiently increase the presence of anaerobic bacteria associated with BV, likely because menstrual bleeding alters the vaginal environment (e.g., increasing microbial diversity and reducing *Lactobacillus* spp.) (Ref. 124).

Hormonal contraceptives, including the combined oral contraceptive pill (COCP) and the hormonal intrauterine device (IUD), function by continuously releasing synthetic oestrogen and/or progestin throughout the menstrual cycle. These hormones primarily inhibit ovulation and increase the viscosity of cervical mucus, thereby reducing the likelihood of sperm penetration and fertilization, and can also affect the VM composition, as one recent systematic review and meta-analysis revealed a strong negative correlation between the use of any hormonal contraceptive (excluding IUDs) and the prevalence, incidence or recurrence of BV (Refs 125, 126). Menopause marks the permanent end of menstruation in women and is clinically diagnosed after 12 consecutive months without a menstrual period. It most commonly occurs around the median age of 51.4 years. It is associated with substantial hormonal shifts, most notably a marked decrease in oestrogen levels, which results in the thinning of the vaginal epithelium, decreased glycogen levels, and an increase in vaginal cavity pH, ultimately leading to a reduction in *Lactobacillus* spp. population (Ref. 127).

### Antibiotic use and over-the-counter hygiene products

Antibiotics are among the most frequently used medications for treating bacterial infections. In the study conducted by Oh et al., oral antibiotic administration in mice led to dysbiosis and an alteration in the composition of vaginal commensal bacteria, thereby enhancing the mice’s susceptibility to mucosal HSV-2 infection (Ref. 128). Karpinets et al. found that oral treatment with broad-spectrum antibiotics in mice altered both the composition and diversity of the gut and vaginal microbiomes, which subsequently influenced cervical cancer progression in an orthotopic model. While antibiotics reduced microbial richness and diversity in the gut, they increased both in the vagina. Beneficial taxa such as *Bacteroides*, *Ruminococcaceae* and *Lachnospiraceae* were enriched vaginally. In contrast, potential pathogens like *Proteobacteria* declined (Ref. 129). While antibiotic therapy effectively decreases the prevalence of *G vaginalis* and other species associated with BV, the post-treatment microbiota is predominantly composed of *L. iners*, rather than the more protective species such as *L. crispatus* and *L. jensenii* (Ref. 130). Ahrens et al. tracked the vaginal microbiota over 26 days in women treated for STIs or BV, comparing them to those from healthy controls. Treatment with metronidazole shifted the microbiota from CST-IV to CST-III in 50% of patients, while azithromycin and tetracyclines had minimal effect on the microbiota structure. *G. vaginalis* persisted in 30% of BV cases, and *Ureaplasma parvum* was often detected even after azithromycin treatment (Ref. 131). On the other hand, Mayer et al. reported a rapid and significant shift in the vaginal microbiota after treatment for BV using specific qPCRs. They observed that Leptotrichia/Sneathia and Megasphaera were quickly cleared, and a substantial reduction in *G. vaginalis* was noted in all patients. However, since most women were treated with local metronidazole, the higher local concentrations of the drug may account for the observed differences (Ref. 132).

Practices related to personal hygiene, such as vaginal douching or other intravaginal activities (e.g. cleansing or the insertion of products), have the potential to affect the balance and stability of the vaginal microbiota. Various factors, including religious beliefs, cultural norms and the level of education or awareness regarding health often shape the choices individuals make regarding these practices (Ref. 133). Certain over-the-counter vaginal products can influence the vaginal epithelium and may have a cytotoxic effect on the survival of beneficial *Lactobacillus* species in the vaginal environment (Ref. 134). For instance, using soap or shampoo for vaginal cleansing could disrupt the vaginal microbiota, which may increase the risk of vaginal symptoms compared to simply using water for washing (Ref. 135). In a study involving 25 healthy Dutch women with a median age of 24 years, 60% of whom used combined oral contraceptives, the effect of an over-the-counter lactic acid-containing intravaginal douching product on the vaginal microbiota was assessed. While routine vaginal douching did not significantly alter the composition of the vaginal microbiota or pH, its use during menstruation was associated with a 2.6-fold increased risk of vaginal dysbiosis (OR = 2.6; 95% CI: 1.0–6.5; *p* = 0.099). Additionally, menstruation alone was linked to a higher presence of anaerobes associated with BV (OR = 1.7; 95% CI: 1.0–2.8), and douching appeared to promote the occurrence of *C. albicans* infections (Ref. 136). A recent observational study by Brown et al. involving 33 sexually active women found that simply discontinuing vaginal douching did not result in significant alterations to the vaginal microbiota or an increased risk of BV. The study suggested that other external influences, including antibiotic use, lubricants, dietary factors, smoking cessation, and condom use, were more strongly associated with changes in the vaginal microbiota (Ref. 137).

### Sexual activity and semen exposure

Recent studies have explored the potential connection between sexual activity and the vaginal microbiota, revealing that sexual behaviour can affect both its composition and diversity, while also significantly contributing to lower genital tract inflammation and the risk of STIs (Ref. 137). Moreover, various sexual behaviours, such as penile-vaginal intercourse, receptive oral sex, the utilization of sex toys and condom application, may significantly impact the composition of the vaginal microbiota (Ref. 138). In studies examining the effects of sexual intercourse with a stable partner on VM composition, no significant differences were found between women with stable partners and those with multiple partners (Ref. 139). However, women who engaged in penile-vaginal intercourse were more likely to be colonized by a type III community state, with *G. vaginalis* present in a minority of cases (Ref. 139). The results suggest that CST type III, which is dominated by *L. iners*, may not offer protective benefits and could serve as a risk factor for the development of STIs. Additionally, studies propose that *G. vaginalis* is acquired from the penile skin microbiota of a sexual partner, supporting the theory that *G. vaginalis* can be transmitted sexually during unprotected intercourse (Ref. 140). Additionally, prostitution is a notable risk factor for alterations in the vaginal microbiota. It is frequently linked to various sexual practices, multiple partners, inconsistent condom use, and high rates of contraceptive use (Ref. 140). The VM of sex workers typically lacks *Lactobacillus*, with bacterial communities characterized by CST-IV, significantly increasing the risk of acquiring STIs, particularly HIV (Ref. 140).

Exposure to semen during unprotected sexual intercourse, as well as inconsistent or incorrect use of condoms, can contribute to the heterosexual transmission of HIV. This may occur by influencing the inflammatory response and disrupting the natural balance of the vaginal microbiota in the female genital tract, which could increase the risk of infection (Ref. 141). Moreover, semen can influence various physiological and pathological processes in the female genital tract. This includes changes in tissue structure, immune reactions to foreign antigens present in seminal fluid, and an increased risk of bacterial and viral infections, such as HIV (Ref. 141). The presence of microbial communities in semen can trigger immune and inflammatory responses by influencing and potentially disrupting the composition of the vaginal microbiota (Ref. 142). Following unprotected sexual intercourse, the microbial content of semen has been linked to a notable reduction in the levels of naturally dominant *Lactobacillus* species, along with an increase in various bacterial species commonly associated with BV (Ref. 142).

### Modulating the vaginal microbiome to prevent STI-targeted therapies

Emerging evidence suggests that modulation of the vaginal microbiome toward a *Lactobacillus*-dominant state may be associated with improved gynaecological and reproductive health, including reduced risk of vaginitis (such as BV), cervical intraepithelial neoplasia, STIs and preterm birth, highlighting its potential as a therapeutic target in women’s health (Ref. 143). Conventional therapeutic approaches, such as antibiotics and antifungal agents, frequently demonstrate limited efficacy and are associated with substantial rates of disease recurrence (Ref. 144). Potential mechanisms through which exogenous *Lactobacillus* strains may influence the VM include vaginal recolonization, enhanced production or release of lactic acid and other antimicrobial substances, as well as modulation of the local mucosal immune response (Ref. 144). Antibiotics often fail to penetrate BV-associated biofilms effectively, and prolonged use of these agents for recurrent BV has been associated with an increased risk of adverse reactions, antimicrobial resistance and secondary fungal infections, such as candidiasis (Ref. 145). Multiple prospective studies have identified a link between disrupted VM, particularly BV and reduced levels of *Lactobacillus* species, and a heightened risk of acquiring STIs (Ref. 146). Women with BV have been reported to experience higher rates of infections such as HPV, HIV, HSV and pelvic inflammatory disease (PID). Accordingly, BV, a prevalent vaginal infection that is often asymptomatic, may serve as a predisposing factor for the acquisition of STIs. Therefore, eliminating this widespread condition in both developed and developing countries could be considered a key strategy in the primary prevention of STIs (Ref. 146). Various strategies have been proposed to therapeutically modulate the vaginal microbiome, including probiotics, prebiotics, synbiotics and VMT (Ref. 147).

### Probiotics

Probiotics refer to live microorganisms that offer health benefits when administered in adequate amounts (Ref. 148). In the context of vaginal health, they are commonly applied either as preventive or therapeutic agents for vaginal disorders, and often serve as complementary treatments alongside conventional antibiotic or antifungal regimens. While generally regarded as safe, probiotics may occasionally lead to adverse effects, ranging from mild gastrointestinal symptoms to more serious complications such as sepsis or the horizontal transfer of antibiotic resistance genes to host microbiota (Ref. 143). Research on probiotic therapy targeting the vaginal microbiota has predominantly focused on BV, with limited investigation into candidiasis and other forms of dysbiosis (Ref. 143). Several factors complicate the interpretation of studies on probiotic therapy for vaginal health. First, probiotic formulations vary widely in their composition, often including different *Lactobacillus* and non-*Lactobacillus* species. Second, there is limited evidence supporting successful vaginal colonization by administered strains, and many studies fail to distinguish between endogenous and probiotic lactobacilli. Third, inconsistent manufacturing standards and insufficient quality control raise concerns about the purity and potency of commercial probiotic products (Ref. 149). Various vaginal probiotic formulations containing *Lactobacillus* species have been developed and evaluated, but their outcomes have generally been inconsistent (Ref. 150). Several factors may account for the limited effectiveness of these probiotic treatments. In certain instances, the formulations included bacterial strains not typically found in the human vaginal microbiota, as they were developed initially for gastrointestinal applications (Ref. 151). Additionally, some probiotics were administered orally with the assumption that systemic physiological changes would promote a vaginal environment conducive to *Lactobacillus* colonization (Ref. 151). A recent randomized, double-blind, placebo-controlled clinical trial evaluated the effectiveness of Lactin-V, a vaginally administered probiotic containing *L. crispatus.* Administered to women with BV following metronidazole therapy, the probiotic was associated with a 15% reduction in BV recurrence compared to placebo, with recurrence rates of 30% and 45%, respectively (Ref. 152). Due to the crucial role lactobacilli play in preventing infections such as HPV, HIV and HSV – as demonstrated in laboratory and animal studies – there has been increasing interest in developing lactobacilli-based probiotics as potential therapeutic agents for STIs in women. As a result, several investigations and clinical trials have been conducted to assess their efficacy (Ref. 11). Palma *et al*. have further demonstrated the significance of reestablishing eubiosis in managing HPV infections. In their investigation, 117 women with coexisting HPV and either BV or bacterial vaginitis – who had previously received antimicrobial therapy (metronidazole or fluconazole) – were administered *L. rhamnosus* BMX 54 (NORMOGIN®) as vaginal tablets for either a short duration (3 months) or a prolonged period (6 months). Women who underwent the extended probiotic regimen experienced markedly fewer recurrences of bacterial and vaginal infections than those on the shorter course. Additionally, long-term use of the probiotic was associated with improvements in HPV outcomes, including higher rates of resolution of HPV-related cytological abnormalities and increased overall clearance of the virus (Ref. 153). Given that *L. crispatus* M247 is a well-characterized probiotic strain known for its ability to establish itself in the vaginal environment after gastrointestinal transit, it has been suggested that restoring vaginal eubiosis with this strain may enhance host defences against HPV, thereby promoting viral clearance. Supporting this hypothesis, a separate clinical trial demonstrated that a 90-day oral administration of *L. crispatus* M247 led to a shift toward a CST I profile (characterized by *L. crispatus* dominance) in the majority of HPV-positive participants, which correlated with a higher HPV clearance rate. By the conclusion of the trial, participants whose vaginal microbiota remained in CST III or CST IV continued to test positive for high-risk or low-risk HPV. In contrast, only 20% of those with CST I microbiota remained HPV-positive (Ref. 154). Table 2 summarizes the current clinical trials on probiotics, prebiotics, and synbiotics for vaginal flora restoration.

**Table 2.** Summary of clinical trials evaluating probiotic, prebiotic and synbiotic interventions for vaginal health Table 2. long description.

Intervention	Conditions	Enrollment	Phase	Findings	NCT. Id
Drug: *Lactobacillus crispatus* M247	Vaginal flora imbalance, Human Papillomavirus (HPV) infection	90 (estimated)	Phase 4	Daily supplementation with *L. crispatus* M247 is being investigated as a potential approach to restore vaginal microbiota balance in women with HPV infection. The study is still in progress, and results have not yet been released.	NCT05808816
Dietary supplement: *L. crispatus* M247 (Crispact®) Dietary supplement: placebo	HPV infection	150 (estimated)	Not applicable	This multicentre randomized study evaluated daily *L. crispatus* M247 (Crispact®), given orally and as a vaginal lavage, to support vaginal microbiota restoration and HPV clearance. The trial is not yet recruiting, and no results are available.	NCT06245486
Dietary supplement: *L. crispatus* M247 (Crispact®) Dietary supplement: placebo	HPV infection	66 (actual)	Not applicable	This randomized, single-blind multicentre study assessed the effects of daily *L. crispatus* M247 supplementation in women with high-risk HPV infection and low-grade cervical lesions. The trial, completed in 2024, evaluated HPV clearance and vaginal microbiota changes after 4 months compared to placebo. Results are pending publication.	NCT06802809
Device: monnalisa touch® laser therapy Combination product: monnalisa touch® laser therapy + *L. crispatus* M247 (Crispact®)	Atrophic vulvovaginitis	75 (estimated)	Not applicable	This randomized, single-blind, multicentre study was designed to evaluate the efficacy and safety of *L. crispatus* M247 combined with vaginal laser therapy in postmenopausal women with atrophic vaginitis. The trial has not yet started recruiting participants, and no results are currently available.	NCT06978907
Dietary supplement: STP4 (*L. plantarum* LP115, *L. helveticus* LA25, *Lactobacillus rhamnosus* LRH10, *Lactobacillus paracasei* LPC12, *Lactobacillus fermentum* LF26, *Lactobacillus delbrueckii* subsp. lactis, LDL114. Dietary supplement: placebo (product without probiotics)	Vaginal diseases	80 (actual)	Not applicable	This interventional trial evaluated the efficacy of the multi-strain probiotic supplement STP4 versus placebo in women with vaginal diseases. The study aimed to assess improvements in vaginal health outcomes. Recruitment has been completed; however, detailed results have not yet been published.	NCT03940612
Dietary supplement: probiotic (antibiotic plus probiotic): metronidazole plus prOVag (containing lactic acid bacteria) Dietary supplement: Placebo (antibiotic plus placebo)	Bacterial vaginosis (BV), Bacterial vaginitis	594 (Actual)	Not applicable	This randomized placebo-controlled trial showed that oral prOVag probiotic supplementation significantly delayed recurrence of BV and aerobic vaginitis in women, improved vaginal pH, Nugent scores, and increased *Lactobacillus* counts.	NCT01993524
Drug: probiotics (Umeta-Miyue, *L. rhamnosus* GR–1 and *Lactobacillus reuteri* RC–14) and metronidazole Drug: metronidazole vaginal	BV	126 (Actual)	Phase 4	This clinical trial evaluated the adjunctive use of *L. rhamnosus* GR–1 and *L. reuteri* RC–14 probiotics alongside metronidazole in women with BV. The results demonstrated improvement in vaginal microbiota composition and reduction of clinical symptoms, suggesting probiotics may enhance standard treatment outcomes for BV.	NCT03894813
Dietary supplement: oral probiotic product	Vaginal health	37 (actual)	Not applicable	This interventional study assessed the effects of an oral probiotic supplement on vaginal health in participants. However, detailed results are not yet publicly available, and further data is needed to evaluate efficacy.	NCT03543982
Dietary supplement: metronidazole and *L. crispatus* CTV–05, Dietary supplement: metronidazole and placebo	BV	228 (actual)	Phase 2	In this randomized, double-blind, placebo-controlled trial, women with BV were initially treated with oral metronidazole followed by administration of *L. crispatus* CTV–05 (LACTIN-V) or placebo to prevent recurrence. The study demonstrated that LACTIN-V significantly reduced BV recurrence rates compared to placebo (30% versus 45%, respectively; statistically significant). Adherence to the intervention was high in both groups, and participants reported high satisfaction with the ease of use and vaginal health improvement in the LACTIN-V group.	NCT02766023
Drug: LACTIN-V (*L. crispatus* CTV–05) Drug: placebo	BV, HIV infections	45 (actual)	Phase 2	In this trial, vaginal administration of LACTIN-V (*L. crispatus* CTV–05) after metronidazole was safe and well tolerated in women at high risk of HIV. LACTIN-V increased *L. crispatus* dominance and reduced post-treatment increases in activated HIV target cells compared to placebo.	NCT05022212
Biological: LACTIN-V applicator (low dose, medium dose, high dose) Other: placebo applicator (low dose, medium dose, high dose)	BV	12 (actual)	Phase 1	In this randomized trial, 12 healthy, sexually abstinent premenopausal women received vaginal doses of LACTIN-V, a formulation containing *L. crispatus* CTV–05, at three escalating levels (5 × 10^8^, 1 × 10^9^, or 2 × 10^9^ CFU) administered once daily for 5 days. The study aimed to assess the product’s safety, tolerability, and user acceptability. Adverse events were mild to moderate, primarily local and evenly distributed across dose groups, with no serious concerns identified. The formulation was deemed safe, well tolerated, and acceptable via vaginal applicator delivery.	NCT00537576
Drug: LACTIN-V Drug: placebo control substance	BV	40 (estimated)	Phase 2	In this randomized trial, women with BV received vaginal LACTIN-V after metronidazole treatment. LACTIN-V safely colonized 61–78% of women who used it as directed, with only mild or moderate side effects similar to placebo. The product was well tolerated and showed promise for restoring healthy vaginal flora.	NCT00635622
Drug: microbial product FB101 Other: placebo (saline water)	Vaginal flora imbalance	34 (actual)	Not applicable	In this randomized, double-blind, placebo-controlled trial, women with asymptomatic vaginal dysbiosis received FB101, a vaginal microbial immunotherapeutic containing live beneficial *Lactobacillus* strains, for 3 days. The treatment was well tolerated with no serious adverse events. About half of the women showed successful engraftment of healthy *Lactobacillus* strains, and the vaginal microbiome shifted toward a more balanced, *Lactobacillus*-dominated state for up to 8 weeks. Inflammatory markers associated with dysbiosis were also reduced, suggesting FB101 may offer a safe, antibiotic-free approach to restoring vaginal health.	NCT05114031
Drug: *L. capsules* (*L. rhamnosus* GR–1 and *L. reuteri* RC–14) Other: placebo gelatin pill	BV	14 (actual)	Early Phase 1	This completed randomized, double-blind, placebo-controlled trial assessed the effects of vaginal administration of *L. rhamnosus* GR–1 and *L. reuteri* RC–14 in asymptomatic postmenopausal women. The probiotic treatment significantly increased vaginal colonization by beneficial *Lactobacillus* species and elevated lactic acid levels while reducing the abundance of potentially harmful bacteria such as *Atopobium.* Immune profiling demonstrated modulation of host responses, including regulation of pattern recognition receptors like TLR2 and enhancement of epithelial barrier function. The probiotics were well tolerated with no serious adverse events reported. These findings indicate that vaginal probiotics can safely restore a healthy vaginal microbiome and positively influence immune function in postmenopausal women.	NCT02139839
Dietary supplement: probiotics (*Lactobacillus* strains, *L. rhamnosus*/ LbV96, *Lactobacillus jensenii* /LbV 116, *L. crispatus*/ Lbv88, *Lactobacillus gasseri* /LbV 150 N) Dietary supplement: oral lactose placebo	Breast cancer	27 (actual)	Not applicable	This randomized, double-blind study tested oral *Lactobacillus* probiotics in some breast cancer patients undergoing chemotherapy. After 2 weeks, 63% of those receiving probiotics showed improved vaginal microbiota by Nugent score versus 36% in placebo. No side effects were reported. The probiotics were safe and helped improve vaginal flora during chemotherapy.	NCT01723592
Drug: *L. rhamnosus* BMX 54	Vaginal infection	117 (actual)	Phase 2	This randomized, double-blind trial studied some women with BV or vaginitis and HPV infection. Patients received standard treatment plus either short-term (3 months) or long-term (6 months) vaginal *L. rhamnosus* BMX 54. After about 14 months, the long-term probiotic group showed significantly higher resolution of HPV-related cytological abnormalities (79.4% versus 37.5%) and greater HPV clearance (31.2% versus 11.6%) compared to the short-term group. These results suggest that prolonged vaginal application of *L. rhamnosus* BMX 54 helps restore vaginal balance and aids HPV infection clearance.	NCT03372395
Drug: VH–01 vaginal suppository tablet Other: placebo vaginal suppository tablet	Vaginal personal care	200 (actual)	Not applicable	This randomized, double-blind, placebo-controlled trial evaluated the efficacy and user experience of VH–01, a synbiotic vaginal suppository containing *L. crispatus* and prebiotics, in women with self-reported vaginal discomforts such as malodour, discharge, itching, irritation, and dryness. Results demonstrated that within 21 days of treatment, 90% of VH–01 users achieved an optimal vaginal microbiome dominated by *L. crispatus.* The intervention was well-tolerated, safe, and significantly improved vaginal health and quality of life. These findings support VH–01 as a promising therapeutic option for restoring vaginal eubiosis and alleviating symptoms of vaginal dysbiosis.	NCT06236893
Dietary supplement: prebiotic + probiotic supplement (biotin, *Lactobacillus acidophilus*, *Bifidobacterium lactis*, *L. rhamnosus*, *L. crispatus*, *L. gasseri*, *L. reuteri*, *Bacillus subtilis*, PreforPro®, white rice flour, magnesium stearate, silicon dioxide and cellulose) Dietary supplement: active placebo supplement (Biotin, *L. acidophilus*, *B. lactis*, *L. rhamnosus*, *L. crispatus*, *L. gasseri*, *L. reuteri*, *B. subtilis*, Inulin (from chicory root), white rice flour, magnesium stearate, silicon dioxide, & cellulose. Dietary supplement: excipient placebo supplement (white rice flour, magnesium stearate, and cellulose).	BV, Yeast infection	120 (estimated)	Not applicable	This randomized, double-blind, placebo-controlled trial is designed to evaluate the efficacy of a prebiotic and probiotic supplement in alleviating symptoms associated with BV and yeast infections in women. Participants will be randomized to receive either the active supplement or a placebo, taking two capsules daily for 30 days. The study will assess changes in vaginal microbiome composition, symptom improvement, and overall vaginal health. Recruitment has not yet commenced, and study results are pending.	NCT06665126
Dietary supplement: OMNi-BiOTiC® FLORA plus+	Vaginal microbiome	80 (actual)	Not applicable	This completed randomized study evaluated the effect of the probiotic OMNi-BiOTiC® FLORA plus+ (containing *L. crispatus* LBV88, *L. rhamnosus* LBV96, *L. gasseri* LBV150N, and *L. jensenii* LBV116) with a special fibre mixture) on the vaginal microbiota of some infertile women undergoing fertility treatment. The intervention group received daily supplementation for 1 month, which led to a significant increase in beneficial *Lactobacillus* species in the vaginal microbiome compared to controls. These findings suggest that probiotic supplementation may help restore a healthy vaginal ecosystem and potentially improve fertility outcomes. However, detailed peer-reviewed results have not yet been published, so conclusions remain preliminary.	NCT04471116

*Note:* For information about the status of R&D of probiotics, prebiotics and synbiotics, please see www.clincialtrials.gov.

### Prebiotics and synbiotics

From a microbiome perspective*, prebiotics* are defined as nutraceutical compounds that stimulate the growth or activity of probiotic or beneficial endogenous microorganisms (Ref. 155). Due to the established effectiveness of prebiotics in enhancing intestinal health, studies have explored whether these compounds can also promote the growth of vaginal lactobacilli (Ref. 156). Monocultures of various *Lactobacillus* species (*L. crispatus*, *L. vaginalis*, *L. gasseri*, *Lactobacillus johnsonii*, *L. jensenii*, and *L. iners*), along with BV-associated bacteria and *C. albicans*, were examined *in vitro* to evaluate their ability to utilize prebiotics, including lactitol, lactulose, raffinose, and oligofructose. Among these prebiotics, lactulose was found to selectively stimulate the growth of vaginal lactobacilli, particularly *L. crispatus*, while showing no significant effect on BV-associated bacteria or *C. albicans* (Ref. 156). A randomized clinical trial with 39 participants compared galacto-oligosaccharides and *Trifolium pratense* extract to a placebo, following metronidazole treatment for BV. At 16 days, the treatment group showed consistently low Nugent scores (≤3), while 24% of the placebo group had scores >3 (*p* = 0.016). However, recurrence rates at day 84 were similar between groups (11% in treatment versus 19% in placebo). No adverse effects were reported (Ref. 157). The method of administering prebiotics remains controversial, with vaginal applications, including pessaries, creams, and douches, being the most commonly utilized. Prebiotics are generally considered safe, and any adverse effects, such as diarrhoea, bloating and flatulence, are typically attributed to their osmotic effects in the intestines (Ref. 158).

A limitation of prebiotic use is its reliance on the presence of lactobacilli, which are often absent or significantly reduced in conditions of dysbiosis. *Synbiotics*, a combination of prebiotics and probiotics, are based on the concept that prebiotics can enhance the growth and functionality of probiotics (Ref. 159). In a randomized controlled study involving 48 women with recurrent BV, the addition of a probiotic formulation – comprising *L. acidophilus* GLA-14 and *L. rhamnosus* HN001 – along with bovine lactoferrin as an adjunct to metronidazole therapy was found to significantly reduce the recurrence rate of the condition (Ref. 160). In another randomized, double-blind clinical trial, administering a combination of micronutrients and *L. rhamnosus* CAN-1 to 21 women undergoing HIV treatment resulted in increased CD4+ cell counts, fewer hospitalizations, and an overall improvement in quality of life (Ref. 161). In a double-blind, placebo-controlled trial, a synbiotic regimen containing *L. plantarum* and *Pediococcus acidilactici* was found to be safe and produced a modest increase in the CD4+/CD8+ ratio among HIV-positive participants (Ref. 162). In contrast, supplementation with *Lactobacillus casei* Shirota over 12 weeks did not yield significant benefits in individuals living with HIV (Ref. 163).

### Vaginal microbiota transplantation: emerging data and ethical considerations

**Figure 2.**
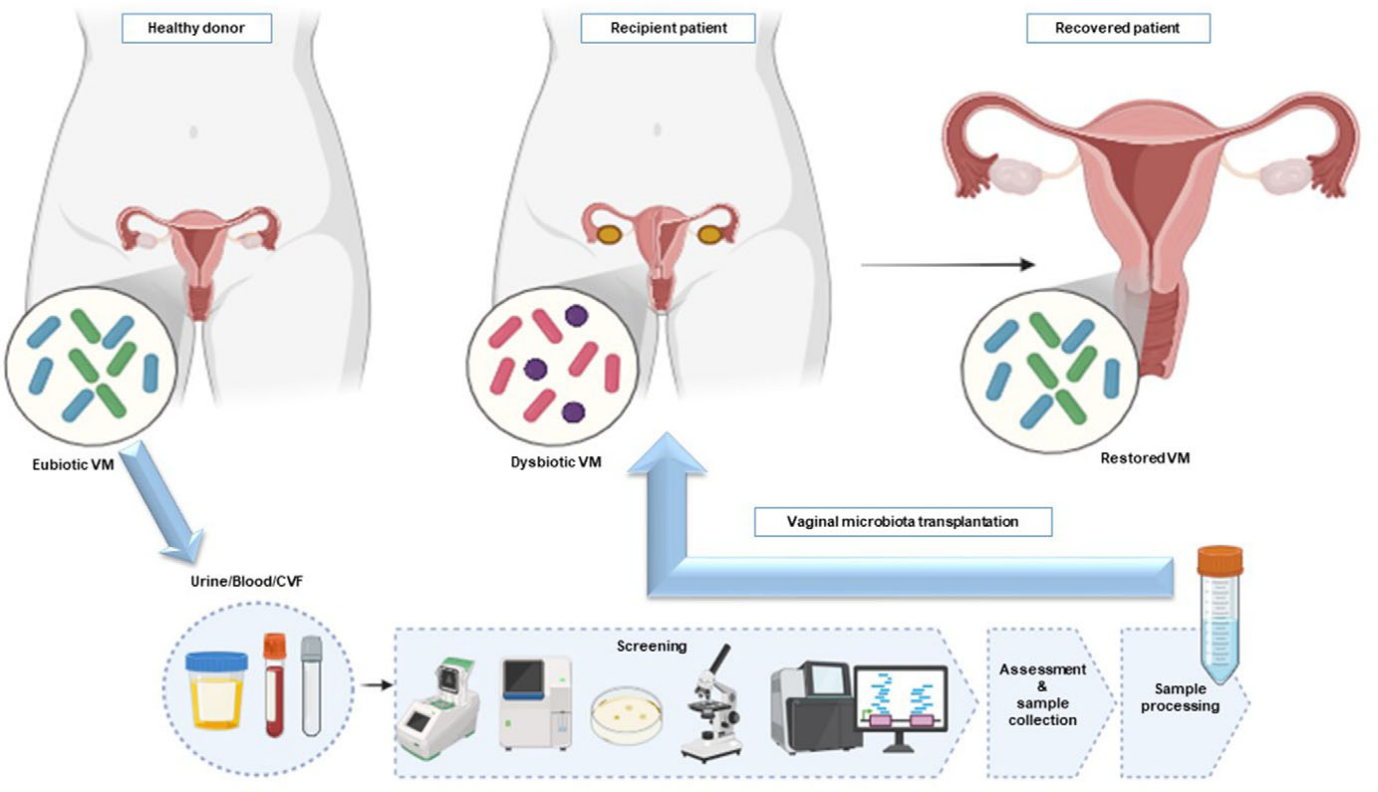
Vaginal microbiota transplantation is an emerging therapeutic strategy aimed at reestablishing a balanced vaginal microbial community and mitigating BV. The primary objective of this intervention is to restore the disrupted microbial homeostasis commonly associated with BV. To begin with, suitable donors undergo comprehensive screening, which involves testing their blood, CVF, and urine. Following this, CVF samples are obtained and prepared under controlled conditions. Finally, the processed CVF, containing a balanced and healthy vaginal microbiota, is introduced into recipients to reestablish a stable vaginal microbial ecosystem and promote normal physiological function. BV: bacterial vaginosis; CVF: cervicovaginal fluid; VM: vaginal microbiome. Figure 2. long description.

VMT involves the transfer of vaginal fluid from healthy donors to recipients, aiming to restore microbial balance (eubiosis) by introducing an entire exogenous bacterial community. In contrast to probiotics, which typically deliver selected bacterial strains, VMT provides a complete and diverse microbiome (Ref. 164) (Figure 2). A 2019 case series was the first to evaluate VMT in five women with recurrent BV unresponsive to antibiotics. Four achieved sustained remission—two after one transplant, two after multiple – with no adverse effects. Successful cases showed *L. crispatus* dominance, while the partial responder had *L. gasseri*, suggesting *L. crispatus* may be key to effective microbiota restoration (Ref. 165). In a recent case study, a woman with a complex obstetric history and pronounced symptomatic vaginal dysbiosis dominated by *Gardnerella* species was successfully managed using VMT without the need for prior antibiotic treatment. Notably, the procedure resulted in the effective engraftment of donor strains, highlighting the potential of antibiotic-free VMT as a promising strategy for restoring vaginal microbial balance in challenging clinical scenarios (Ref. 166). In a double-blinded randomized trial, Wroending et al. showed that VMT without antibiotics effectively restored a healthy VM in women with dysbiosis. Donor microbiota successfully engrafted, the procedure was safe, and no severe adverse effects were observed, supporting VMT as a promising alternative to antibiotics (Ref. 167). The primary risks associated with VMT include the potential transmission of pathogens from donor to recipient, notably antimicrobial-resistant microorganisms that may evade current detection methods. Additionally, the unintentional transfer of sperm present in vaginal fluid poses a risk of unintended pregnancy. Given that the long-term effects of VMT are still unknown, rigorous donor selection criteria and comprehensive screening protocols are essential to ensure safety. A standardized approach for screening universal VMT donors has been proposed to address these concerns (Ref. 168). Future research involving larger cohorts and randomized, placebo-controlled trials is essential to evaluate the efficacy and long-term stability of VMT. Another emerging theoretical approach within microbiome transplantation involves the genetic engineering or manipulation of microorganisms to endow them with targeted properties, such as the ability to produce therapeutic compounds or exert specific health benefits (Ref. 169). Table 3 highlights the current clinical trials on VMT for vaginal flora restoration.

**Table 3.** Overview of clinical trials on vaginal microbiota transplantation (VMT) for vaginal flora restoration Table 3. long description.

Intervention	Condition	Enrollment	Phase	Findings	NCT. Id
Biological: vaginal flora transplant	Recurrent bacterial vaginitis	10 (estimated)	Not applicable	In this small pilot study, five women with recurrent bacterial vaginosis (BV) received vaginal microbiota transplantation (VMT). Four showed complete symptom resolution with restoration of *Lactobacillus*-dominant vaginal microbiota, while one showed partial improvement. No adverse events were reported.	NCT02236429
Biological: vaginal microbiome transplant Drug: oral metronidazole Other: sterile saline	Recurrent bacterial vaginitis	62 (estimated)	Phase 1 Phase 2	This randomized, placebo-controlled trial evaluated the safety and efficacy of VMT in restoring *Lactobacillus* dominance in women with recurrent BV. Preliminary data showed good safety and promising colonization results.	NCT04046900
Biological: vaginal microbiome transplantation Biological: placebo	BV	100 (estimated)	Not applicable	This study is an ongoing randomized, placebo-controlled clinical trial investigating the safety and efficacy of VMT for women suffering from recurrent BV. Preliminary results from earlier related pilot work have shown that VMT can restore a healthy vaginal microbiota and reduce recurrence rates in women who do not respond well to standard antibiotic treatments. However, the final results of this specific trial are not yet published, as the study is still recruiting.	NCT04517487
Biological: vaginal Microbiome transplant Biological: vaginal microbiome transplant placebo Other: no intervention	Vaginal dysbiosis, Vaginal microbiome	302 (actual)	Not applicable	This ongoing trial aims to evaluate VMT efficacy in restoring vaginal microbiome diversity and improving symptoms in women with vaginal dysbiosis. Preliminary data indicate improvement in microbiome composition and clinical outcomes.	NCT04855006

*Note:* For information about the status of R&D of VMT, please see www.clinicaltrials.gov.

## Conclusion

The vaginal microbiome is a dynamic and diverse microbial ecosystem that changes in response to factors such as age, hormonal status, pregnancy, and menstrual cycle phase. Among the five CSTs identified, CST-I to CST-III and CST-V are dominated by *Lactobacillus* species. Several protective mechanisms are provided by the vaginal microbiota, primarily by *Lactobacillus* species, including the production of H₂O₂, lactic acid and bacteriocins, which exert broad-spectrum antimicrobial activity against STI pathogens such as *N. gonorrhoeae* and *C. albicans.* In addition, some *Lactobacillus* spp. inhibit colonization of STI pathogens through nutrient competition and the formation of protective biofilms. These biofilms involve intra- and interspecies competition, thereby limiting and inhibiting STI pathogen growth. *Lactobacilli*, especially *L. crispatus* and *L. gasseri*, which are dominant in CST-I to CST-III, modulate immune responses by reducing inflammation through suppression of pro-inflammatory cytokines and increasing anti-inflammatory cytokines, as well as enhancing mucosal defences. Vaginal dysbiosis increases susceptibility to STIs such as HIV, HPV, *C. trachomatis*, and *N. gonorrhoeae.* The significant risk factors contributing to vaginal dysbiosis are sexual activity, antibiotic use, hormonal fluctuations, immunosuppression, lack of personal hygiene, ageing and lifestyle factors. Antibiotic therapy can disrupt vaginal microbiota and cause overgrowth of less protective species like *L. iners* rather than the more protective species such as *L. crispatus* and *L. jensenii.* Certain sexual behaviours, such as homosexual or bisexual activities, also significantly raise the likelihood of developing a shift towards CST-IV and acquiring STIs, particularly HIV. Recent evidence indicates that modulation of the vaginal microbiome toward a Lactobacillus-dominant state has the potential to reduce the risks of STIs. Hence, therapeutic strategies aimed at enhancing the vaginal microbiome, such as probiotics, prebiotics, synbiotics and VMT, have been proposed to limit STI transmission. Among the prebiotics, lactulose exhibits selective stimulation of vaginal lactobacilli growth, particularly *L. crispatus.* However, synbiotics showed better activity in improving the CD4+/CD8+ ratio in HIV-positive women. Also, VMT, as a novel therapeutic approach and a promising alternative to antibiotics, effectively restored a healthy vaginal microbiome in women with dysbiosis. However, safety concerns such as pathogen transmission resistant to antibiotics, the risk of unintended pregnancy, highlight the need for standardized donor screening and further research.

## Data Availability

Data sharing is not applicable to this article, as no datasets were generated during the current study.

## Long descriptions

### Table 1. Long description

The table is organized into ten columns representing different C S T categories and sub-types.

* C S T Roman numeral I: Divided into C S T-I A and C S T-I B. Both are dominated by Lactobacillus crispatus. C S T-I A has a lower abundance of Lactobacillus acidophilus and Lactobacillus iners with lower diversity. C S T-I B has a higher abundance of L. acidophilus and higher diversity.

* C S T Roman numeral II: Dominated by Lactobacillus gasseri.

* C S T Roman numeral III: Divided into C S T-III A and C S T-III B. Both are dominated by Lactobacillus iners. C S T-III B is specifically noted for being co-dominated by L. acidophilus and non-Lactobacillus species.

* C S T Roman numeral IV: Divided into four sub-groups. Sub-group A is dominated by Candidatus Lachnocurva vaginae with moderate Gardnerella vaginalis. Sub-group B is dominated by Gardnerella vaginalis and moderate Atopobium vaginae. Sub-group C 0 is dominated by Prevotella. Sub-group C 1, C 2, C 3, C 4 contains high levels of Streptococcus species, Enterococcus species, Bifidobacterium species, or Staphylococcus species isolates.

* C S T Roman numeral V: Dominated by Lactobacillus jensenii.

Navigate back to Table 1..

### Figure 1. Long description

The diagram is divided into two main sections under a central illustration of the female reproductive system.

Panel A, titled Dysbiosis of vaginal microbiota, is on the left. At the top, a blue downward arrow indicates a decrease in antimicrobial byproducts and Lactic acid. This leads to Pathogens invasion and an Inflammatory response, resulting in an Imbalance V M. Below this, C S T I I I / I V diverse microbial communities are shown. A dashed arrow indicates mucus degradation and barrier disruption. The vaginal epithelium is labeled as Disrupted vaginal epithelium, covered by a layer of Thin mucus. Pathogens are shown forming a Polymicrobial biofilm on the surface. Blue and red arrows indicate a decrease in Estrogen and Glycogen, and an increase in Vaginal discharge and p H greater than 4.5.

Panel B, titled Normal vaginal microbiota, is on the right. At the top, a red upward arrow indicates an increase in Antimicrobial byproducts such as Bacteriocin and H sub 2 O sub 2. These byproducts are shown inhibiting pathogens. Below this, the Lactobacillus Domaine C S T I / I I / V is shown. Metabolic pathways show Pyruvate and Maltose converting to Lactic Acid, which is marked with a red upward arrow. The epithelium is labeled as Thick vaginal epithelium, covered by Thick mucus. Blue and red arrows indicate an increase in Estrogen and Glycogen, and a decrease in p H to less than 4.5.

Navigate back to Figure 1..

### Table 2. Long description

The table consists of six columns: Intervention, Conditions, Enrollment, Phase, Findings, and N C T dot Id. It lists 19 separate clinical trials.

* Row 1: Lactobacillus crispatus M 2 4 7 for vaginal flora imbalance and H P V. Enrollment 90. Phase 4. Study in progress.

* Row 2: L. crispatus M 2 4 7 (Crispact) for H P V. Enrollment 150. Not applicable phase. Not yet recruiting.

* Row 3: L. crispatus M 2 4 7 (Crispact) for H P V. Enrollment 66. Not applicable phase. Completed in 2024, results pending.

* Row 4: Monnalisa touch laser therapy plus L. crispatus M 2 4 7 for atrophic vulvovaginitis. Enrollment 75. Not applicable phase. Not yet recruiting.

* Row 5: S T P 4 multi-strain probiotic for vaginal diseases. Enrollment 80. Not applicable phase. Recruitment completed, results pending.

* Row 6: Metronidazole plus pr O Vag for B V and bacterial vaginitis. Enrollment 594. Not applicable phase. Showed significant delay in B V recurrence and improved Nugent scores.

* Row 7: Umeta-Miyue (L. rhamnosus G R-1 and L. reuteri R C-14) plus metronidazole for B V. Enrollment 126. Phase 4. Demonstrated improved microbiota composition.

* Row 8: Oral probiotic product for vaginal health. Enrollment 37. Not applicable phase. Results not publicly available.

* Row 9: Metronidazole and L. crispatus C T V-05 (L A C T I N-V) for B V. Enrollment 228. Phase 2. Significantly reduced B V recurrence (30 percent versus 45 percent).

* Row 10: L A C T I N-V for B V and H I V infections. Enrollment 45. Phase 2. Safe and reduced activated H I V target cells.

* Row 11: L A C T I N-V applicator (escalating doses) for B V. Enrollment 12. Phase 1. Safe and well tolerated at doses up to 2 times 10 super 9 C F U.

* Row 12: L A C T I N-V for B V. Enrollment 40. Phase 2. Safely colonized 61 to 78 percent of users.

* Row 13: Microbial product F B 1 0 1 for vaginal flora imbalance. Enrollment 34. Not applicable phase. Shifted microbiome toward Lactobacillus-dominated state for up to 8 weeks.

* Row 14: L. rhamnosus G R-1 and L. reuteri R C-14 for B V in postmenopausal women. Enrollment 14. Early Phase 1. Increased colonization and reduced Atopobium.

* Row 15: Multi-strain Lactobacillus probiotics for breast cancer patients. Enrollment 27. Not applicable phase. 63 percent showed improved microbiota versus 36 percent in placebo.

* Row 16: L. rhamnosus B M X 54 for vaginal infection and H P V. Enrollment 117. Phase 2. Long-term use (6 months) showed 79.4 percent resolution of cytological abnormalities.

* Row 17: V H-01 synbiotic suppository for vaginal personal care. Enrollment 200. Not applicable phase. 90 percent of users achieved optimal microbiome within 21 days.

* Row 18: Prebiotic plus probiotic supplement for B V and yeast infection. Enrollment 120. Not applicable phase. Recruitment not yet commenced.

* Row 19: O M N i-B i O T i C F L O R A plus for vaginal microbiome in infertile women. Enrollment 80. Not applicable phase. Significant increase in beneficial Lactobacillus after 1 month.

Navigate back to Table 2..

### Figure 2. Long description

The diagram is organized into three main stages across the top and a processing workflow at the bottom.

Top-Left: Healthy donor. An anatomical illustration shows a healthy reproductive system. A circular inset labeled Eubiotic V M displays a balanced mix of green and blue rod-shaped bacteria. A blue arrow points down to a collection of Urine forward slash Blood forward slash C V F samples in a cup and test tubes.

Bottom Row: The samples move right into a Screening box containing lab equipment, a petri dish, and a microscope. This leads to an Assessment and sample collection stage, followed by a Sample processing stage where a final purified blue liquid is shown in a conical tube.

Center-Top: Recipient patient. The anatomical illustration shows yellow-shaded areas indicating infection. A circular inset labeled Dysbiotic V M shows a mix of pink rods and purple spheres. A large blue L-shaped arrow labeled Vaginal microbiota transplantation points from the processed sample tube up toward this patient.

Top-Right: Recovered patient. A black arrow points from the recipient to the final stage. The anatomical illustration shows a healthy state, and the circular inset labeled Restored V M shows the return of the green and blue rod-shaped bacteria.

Navigate back to Figure 2..

### Table 3. Long description

The table consists of six columns and four rows of data.

* Row 1: Intervention is Biological vaginal flora transplant. Condition is Recurrent bacterial vaginitis. Enrollment is 10 estimated. Phase is Not applicable. Findings state that in a pilot study of five women with recurrent B V, four showed complete symptom resolution and restoration of Lactobacillus-dominant microbiota with no adverse events. N C T Id is N C T 0 2 2 3 6 4 2 9.

* Row 2: Interventions include Biological vaginal microbiome transplant, Drug oral metronidazole, and Other sterile saline. Condition is Recurrent bacterial vaginitis. Enrollment is 62 estimated. Phase is Phase 1 and Phase 2. Findings indicate this randomized placebo-controlled trial showed good safety and promising colonization results for restoring Lactobacillus dominance. N C T Id is N C T 0 4 0 4 6 9 0 0.

* Row 3: Interventions are Biological vaginal microbiome transplantation and Biological placebo. Condition is B V. Enrollment is 100 estimated. Phase is Not applicable. Findings describe an ongoing trial where earlier pilot work showed V M T can restore healthy microbiota and reduce recurrence, though final results are not yet published. N C T Id is N C T 0 4 5 1 7 4 8 7.

* Row 4: Interventions include Biological vaginal microbiome transplant, Biological placebo, and Other no intervention. Condition is Vaginal dysbiosis and Vaginal microbiome. Enrollment is 302 actual. Phase is Not applicable. Findings note an ongoing trial where preliminary data indicate improvement in microbiome composition and clinical outcomes. N C T Id is N C T 0 4 8 5 5 0 0 6.

Navigate back to Table 3..
